# Benfotiamine, a synthetic S-acyl thiamine derivative, has different mechanisms of action and a different pharmacological profile than lipid-soluble thiamine disulfide derivatives

**DOI:** 10.1186/1471-2210-8-10

**Published:** 2008-06-12

**Authors:** Marie-Laure Volvert, Sandrine Seyen, Marie Piette, Brigitte Evrard, Marjorie Gangolf, Jean-Christophe Plumier, Lucien Bettendorff

**Affiliations:** 1 Center for Cellular and Molecular Neurobiology, University of Liège, Avenue de l'Hôpital, 1, 4000 Liège, Belgium; 2 Laboratory of Pharmaceutical Technology, Department of Pharmacy, University of Liège, Avenue de l'Hôpital, 1, 4000 Liège, Belgium

## Abstract

**Background:**

Lipid-soluble thiamine precursors have a much higher bioavailability than genuine thiamine and therefore are more suitable for therapeutic purposes. Benfotiamine (S-benzoylthiamine O-monophosphate), an amphiphilic S-acyl thiamine derivative, prevents the progression of diabetic complications, probably by increasing tissue levels of thiamine diphosphate and so enhancing transketolase activity. As the brain is particularly sensitive to thiamine deficiency, we wanted to test whether intracellular thiamine and thiamine phosphate levels are increased in the brain after oral benfotiamine administration.

**Results:**

Benfotiamine that is practically insoluble in water, organic solvents or oil was solubilized in 200 mM hydroxypropyl-β-cyclodextrin and the mice received a single oral administration of 100 mg/kg. Though thiamine levels rapidly increased in blood and liver to reach a maximum after one or two hours, no significant increase was observed in the brain. When mice received a daily oral administration of benfotiamine for 14 days, thiamine derivatives were increased significantly in the liver but not in the brain, compared to control mice. In addition, incubation of cultured neuroblastoma cells with 10 μM benfotiamine did not lead to increased intracellular thiamine levels. Moreover, in thiamine-depleted neuroblastoma cells, intracellular thiamine contents increased more rapidly after addition of thiamine to the culture medium than after addition of benfotiamine for which a lag period was observed.

**Conclusion:**

Our results show that, though benfotiamine strongly increases thiamine levels in blood and liver, it has no significant effect in the brain. This would explain why beneficial effects of benfotiamine have only been observed in peripheral tissues, while sulbutiamine, a lipid-soluble thiamine disulfide derivative, that increases thiamine derivatives in the brain as well as in cultured cells, acts as a central nervous system drug. We propose that benfotiamine only penetrates the cells after dephosphorylation by intestinal alkaline phosphatases. It then enters the bloodstream as S-benzoylthiamine that is converted to thiamine in erythrocytes and in the liver. Benfotiamine, an S-acyl derivative practically insoluble in organic solvents, should therefore be differentiated from truly lipid-soluble thiamine disulfide derivatives (allithiamine and the synthetic sulbutiamine and fursultiamine) with a different mechanism of absorption and different pharmacological properties.

## Background

It is well known that thiamine deficiency results in neurological disorders such as beriberi or Wernicke-Korsakoff syndrome [1]. It is generally assumed that the symptoms arise from decreased activity of thiamine diphosphate (ThDP) – dependent enzymes such as transketolase and pyruvate and oxoglutarate dehydrogenases, with subsequent impairment of carbohydrate metabolism in the brain. It is not known to what extent, if any, the decrease in other thiamine derivatives such as thiamine triphosphate (ThTP, [2]) and the newly discovered adenosine thiamine triphosphate (AThTP, [3]) are involved in the appearance of these symptoms.

In a number of diseases, beneficial effects of the administration of free, unphosphorylated thiamine have been reported. Thus, high-dose thiamine therapy reversed the symptoms of Wernicke's encephalopathy [1] and prevented incipient diabetic nephropathy [4] and diabetic dyslipidaemia [5] in experimental diabetes in the rat. Other reports suggested that thiamine may protect against free-radical mediated neurotoxicity [6] and that it may have a cytoprotective effect on cultured neonatal rat cardiomyocytes under hypoxic insult [7]. These protective effects may be due to increased tissue ThDP levels after thiamine treatment, but effects mediated by other phosphorylated thiamine derivatives such as ThTP and AThTP cannot be ruled out.

**Figure 1:**
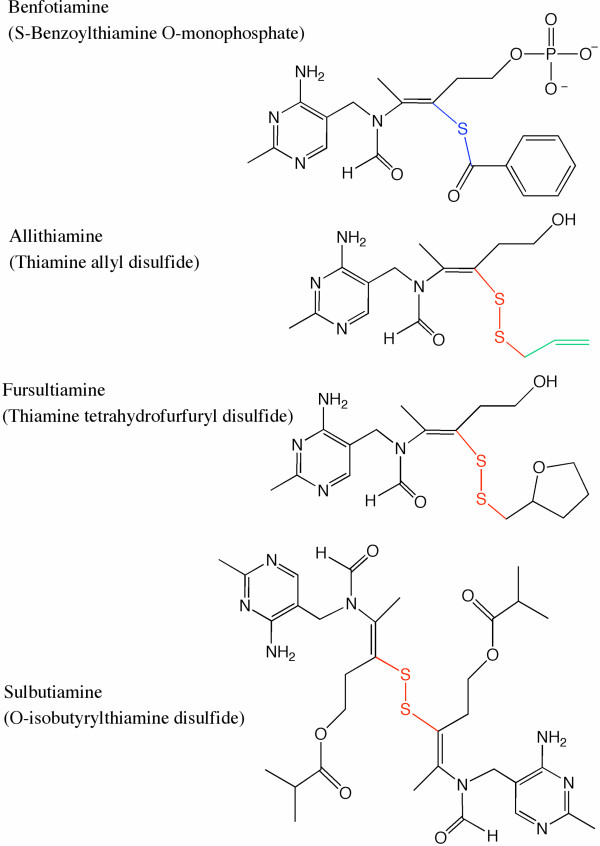
**Structures of the S-acyl derivative benfotiamine and the disulfide compounds allithiamine, fursultiamine and sulbutiamine**. The thioester bond in benfotiamine is indicated in blue, while the disulfide bond in allithiamine, fursultiamine and sulbutiamine is drawn in red. The allyl group in allithiamine is indicated in green.

It is known that free thiamine is transported across plasma membranes by high affinity carriers [8], but the rate of transport is generally slow. For that reason, a variety of lipophilic thiamine derivatives have been synthesized. These compounds can easily diffuse through plasma membranes thus bypassing the rate-limiting transport system required for free thiamine. Once incorporated into the cells, these lipophilic derivatives can be rapidly converted to thiamine through enzymatic or non-enzymatic processes. The first lipophilic thiamine derivative was isolated from garlic (*Allium sativum*) extracts in the early 1950s [9]. It is an allyl disulfide derivative called allithiamine (Fig. 1). Since then, several analogs of this molecule were synthesized with the hope that they would be better absorbed and have a higher bioavailability than thiamine hydrochloride or mononitrate [10,11]. These lipophilic disulfides are often referred to as "allithiamines", in our opinion an improper denomination as they are synthetic molecules not present in *Allium *species and do not possess any allyl group.

Presently, two lipophilic disulfide derivatives are used as therapeutic agents: thiamine tetrahydrofurfuryl disulfide ("fursultiamine", Fig. 1) [12] and O-isobutyrylthiamine disulfide ("sulbutiamine", Fig. 1) [13]. Sulbutiamine turned out to be a psychotropic drug prescribed for the symptomatic treatment of functional asthenias [13]. It was found that chronic treatment with sulbutiamine (52 mg/kg, i.p.) increases thiamine, ThMP, ThDP and ThTP levels in the rat brain as well as in peripheral tissues [14]. Concerning fursultiamine, it is not clear whether it has specific effects on brain function [12] but it exerts a positive inotropic effect in heart muscle [15-17].

S-benzoylthiamine O-monophosphate ("benfotiamine", Fig. 1) is a third derivative with better bioavailability than thiamine. In contrast to the above-mentioned derivatives it is not a disulfide but an S-acyl derivative. It prevents the development and the progression of diabetic complications [18-23]. It was suggested that treatment with benfotiamine blocks three major pathways (the hexosamine pathway, the advanced glycation end product formation pathway and the diacylglycerol-protein kinase pathway) of hyperglycemic damage, probably by removal of glyceraldehyde 3-phosphate and fructose 6-phosphate through activation of the pentose phosphate enzyme transketolase [18]. Other beneficial effects of benfotiamine were the reduction of glucose toxicity [19,20], alleviation of diabetes-induced cerebral oxidative damage [21], acceleration of the healing of ischemic diabetic limbs in mice [22] and rescue of cardiomyocyte contractile dysfunction in experimental diabetes mellitus [23].

Whilst different studies show that administration of benfotiamine leads to higher thiamine blood levels than administration of water-soluble thiamine [10,24-27], practically no information is available concerning its effects on the brain. The aim of the present study was to check whether oral administration of benfotiamine increases the levels of thiamine and its phosphorylated derivatives in the brain of mice.

## Results

### Acute experiment

**Figure 2:**
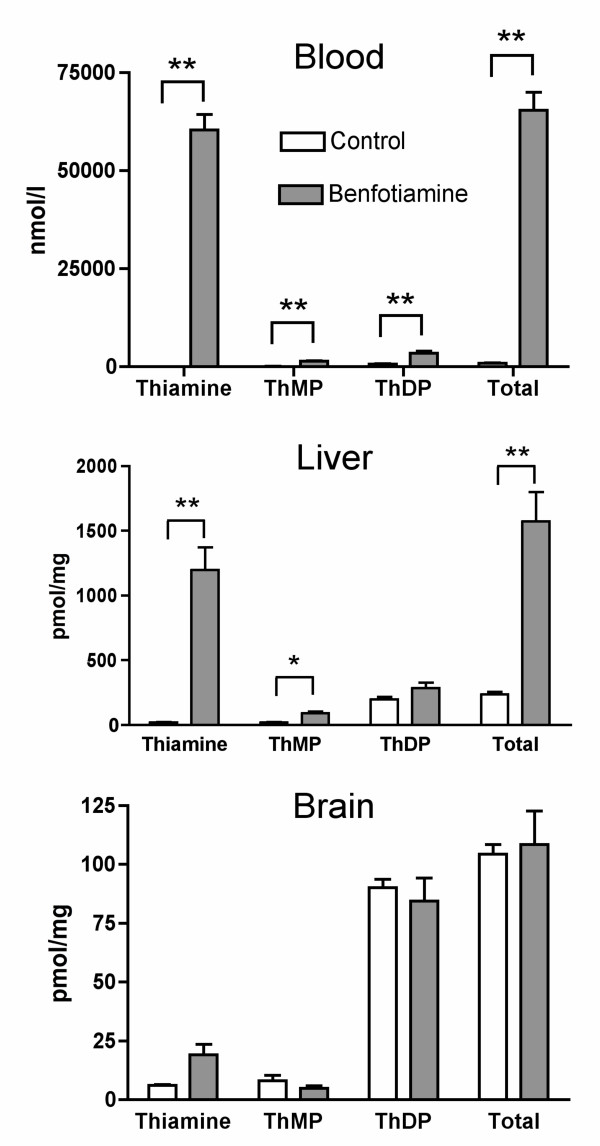
**Contents of thiamine, ThMP, ThDP and total thiamine in the blood, the liver and the brain of mice (strain sv129) 1 hour after a single oral administration of 100 μl of 200 mM HP-b-CD or benfotiamine (100 mg/kg) in approximately 100 μl of a 200 mM HP-b-CD solution**. The data were analyzed by MANOVA (Wilk's Lambda, p = 0.0002, 0.0145 and 0.27 for blood, liver and brain respectively) followed by ANOVA for each thiamine compound (*, p < 0.05; **, p < 0.01). The results are expressed as mean ± SEM for 4 animals in each group.

As previous studies do not mention the use of a specific carrier for the gavage of mice with benfotiamine [18,23], we first suspended benfotiamine in water and administered it by force-feeding (100 μl containing a dose equal to 100 mg of benfotiamine per kg), using a syringe with a tube that was inserted into the stomach. We sacrificed the animals after 1 hour and we determined thiamine derivatives in the blood. Thiamine levels proved to be highly variable: in some animals thiamine levels were up to 10 times higher than in the controls, while in others they were hardly increased. Benfotiamine is very sparingly soluble in water (at least at pH < 8.0) and it is possible that in some cases, either most of the material remained attached to the syringe or the force-feeding tube or it was eliminated with the bolus. Therefore, we tested different hydrophobic solvents such as oil (Mineral oil, Sigma M5904) and organic solvents (ethanol, octanol, dimethyl sulfoxide), but no significant solubility was observed. Finally, we obtained a homogenous solution of benfotiamine by forming inclusion complexes in a 200 mM hydroxypropyl-β-cyclodextrin (HP-β-CD) solution that was used for force-feeding. This time, the inter-animal variability was reasonable (25%, S.E.M/mean × 100, n = 12) and we found that, one hour after the administration, thiamine, ThMP and ThDP levels in the blood were increased by a factor of respectively 822, 14 and 4.7 (Fig. 2). Highly significant increases of free thiamine and ThMP were also observed in the liver, but not in the brain.

### Pharmacokinetics

**Figure 3:**
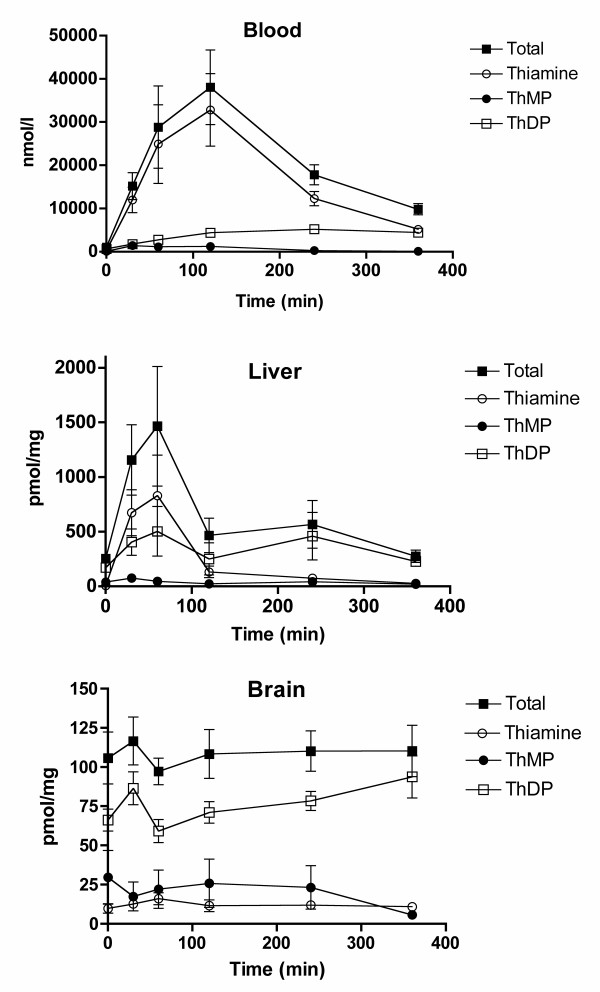
**Time-dependent tissue content of thiamine derivatives after a single oral administration of benfotiamine (100 mg/kg) in approximately 100 μl of a 200 mM HP-β-CD solution in mice (strain C57B6)**. The results are expressed as mean ± SEM for 6 – 7 animals for each time point.

First, we measured tissue levels of thiamine, ThMP and ThDP for up to 6 hours after a single oral administration of benfotiamine (100 mg/kg in a HP-β-CD solution). In the blood, thiamine levels rapidly increased, reaching a maximum after 120 min and slowly decreasing thereafter (Fig. 3). ThDP levels steadily increased before reaching a maximum after 4 hours. For ThMP, the highest level was already reached after 30 min and it slowly decreased during the next hours. In the liver, all derivatives peaked after 1 hour before decreasing to near normal levels after 2 hours. No significant variations were observed in the brain. Total thiamine content (per mg of protein) was about twice higher in the liver than in the brain before benfotiamine administration. One hour after the administration, the ratio was over 10. However, liver thiamine decreased rather fast after 1 – 2 hours. In most samples, we also observed the presence of ThTP and AThTP, but the levels of these compounds in the liver and the brain were not influenced by the treatment (not shown).

### Chronic experiment

**Figure 4:**
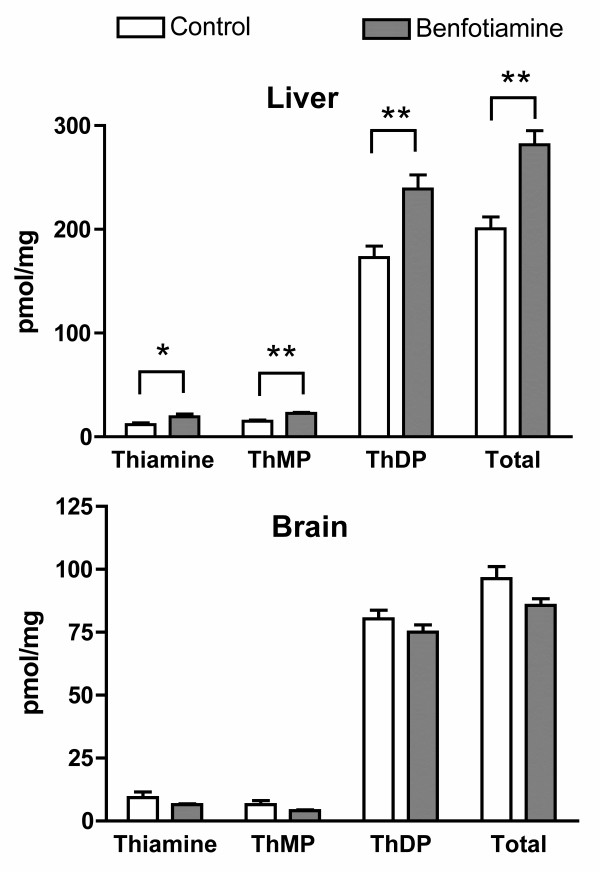
**Contents of thiamine, ThMP, ThDP and total thiamine in the liver and the brain of mice (strain sv129) after daily oral administration of 200 mM HP-β-CD (8 animals) or benfotiamine (100 mg/kg) in approximately 100 μl of a 200 HP-β-CD solution (10 animals) for 14 days**. The data (mean ± SEM) were analyzed by MANOVA (Liver : Wilk's Lambda = 0.24, p = 0.0061; Brain : Wilk's Lambda = 0.779, p = 0.48) followed by an ANOVA for each thiamine compound (*, p < 0.05; **, p < 0.01).

As no increase in brain thiamine levels was observed after a single administration of benfotiamine, we decided to test a longer treatment. The animals received a daily oral administration of either 100 μl of a solution of benfotiamine (100 mg/kg) in 200 mM HP-β-CD or 100 μl of a 200 mM HP-β-CD solution for 14 days and were sacrificed 24 hours later. After treatment with benfotiamine, the levels of free thiamine, ThMP and ThDP were increased in the liver but not in the brain (Fig. 4). Again, the small amounts of ThTP and AThTP found did not depend on the conditions of treatment.

### Cell culture experiments

**Figure 5:**
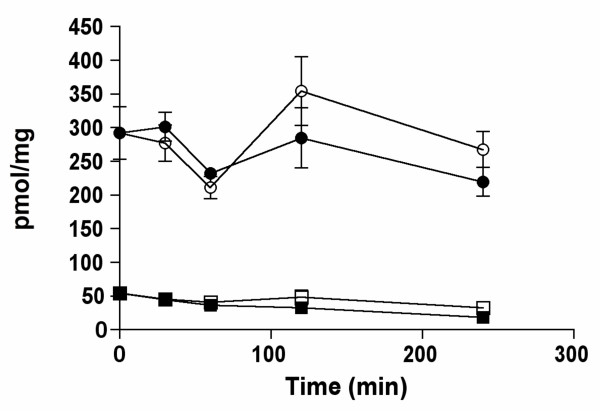
**Contents of total (○, ●) and unphosphorylated (□, ■) thiamine during incubation of Neuro2a cells in the presence of 10 μM thiamine (○, □) or benfotiamine (●, ■)**. The data are expressed as mean ± SEM for 4 experiments.

As treatment with benfotiamine did not yield elevated brain thiamine levels, we wanted to test whether this molecule is able to easily cross cell membranes in cultured cells. We chose mouse neuroblastoma cells (Neuro 2a) as we have previously characterized these cells with respect to thiamine transport [8,28], metabolism [29] and deficiency [30]. The thiamine concentration in the commercial Dulbecco's modified Eagle's medium (DMEM) is 10 μM [8]. Therefore, once the cells were nearly confluent, DMEM was replaced by a saline containing 10 μM of either thiamine or benfotiamine. After incubation for up to 4 h at 37°C, the thiamine content of the cells was determined. We did not observe any increase in intracellular thiamine after incubation with benfotiamine and there were no significant differences between the thiamine and benfotiamine groups (Fig. 5). This is in sharp contrast with previous experiments where incubation of the same cell line with 10 μM sulbutiamine under the same conditions led to a 10-fold increase in intracellular free (unphosphorylated) thiamine within 2 hours [29].

**Figure 6:**
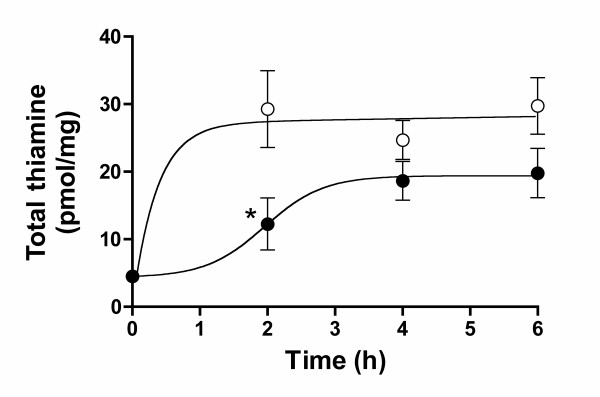
**Effect of thiamine and benfotiamine on the total thiamine content in thiamine-depleted Neuro 2a cells**. The cells were grown for 8 days in thiamine deficient DMEM medium containing 5% fetal calf serum, before addition of thiamine (1 μM, ○) or benfotiamine (1 μM, ●) and intracellular thiamine derivatives were determined at the times indicated. The data (mean ± SEM, n = 3) were analyzed by two-way ANOVA followed by the Bonferroni post-test for comparison of the benfotiamine and the thiamine groups at different times (*, p < 0.05).

As mentioned above, the thiamine concentration in most commercial culture media is about 10 μM, one or two orders of magnitude higher than the *K*_m _for the high affinity thiamine transport present in most cells [8,28,29]. Cells grown under those conditions are thus saturated with thiamine. Therefore, we wanted to test whether benfotiamine had any effect on intracellular thiamine levels in Neuro 2a cells previously thiamine-depleted. The cells were grown for 8 days in a medium containing about 7 nM thiamine. Then either benfotiamine (1 μM) or thiamine (1 μM) was added (Fig. 6). After 2 hours in the presence of thiamine, intracellular thiamine already reached a maximum, while with benfotiamine an important lag period was observed. This experiment confirms that thiamine enters Neuro 2a cells more rapidly than benfotiamine. The lag period can be explained if benfotiamine is first dephosphorylated by ecto-phosphatases to the lipidsoluble S-benzoylthiamine that can then enter the cells (see below).

## Discussion

### Mechanism of action of benfotiamine

In 1961, Wada et al. reported the physicochemical properties of benfotiamine and its possible use as a therapeutic agent [31]. Benfotiamine is more easily absorbed by the body and oral administration results in higher thiamine and ThDP blood levels in animals than an equivalent dose of thiamine. A few years, later Shindo and coworkers [32-35] studied in more detail the mechanism of absorption and the metabolic fate of benfotiamine in animal tissues. Their results suggested that benfotiamine (given orally) is first dephosphorylated to S-benzoylthiamine by the ecto-alkaline phosphatase present in the brush borders of intestinal mucosal cells. The more lipophilic S-benzoylthiamine then diffuses through the membranes of intestinal and endothelial cells and appears in the venous mesenteric blood. A significant part of S-benzoylthiamine is captured by erythrocytes [34] and converted to free thiamine through a slow non-enzymatic transfer of the S-benzoyl group to SH groups of glutathione. In the liver, the remainder can be enzymatically hydrolyzed to thiamine and benzoic acid by thioesterases present in the hepatocytes. On the other hand, thiamine disulfide derivatives require a reduction either enzymatically in the liver by glutathione or non enzymatically in blood by glutathione and possibly other substrates [36]. In the present work, we show that after oral administration of benfotiamine to mice, free thiamine appears in the liver at a fast rate, reaching a maximum after one hour, while in the blood the maximum is reached only after two hours (Fig. 3). We therefore propose that most of the S-benzoylthiamine present in the mesenteric blood is captured by the liver and transformed into thiamine. The excess thiamine formed is then rapidly released into the blood stream, as shown by the fast decrease of thiamine content in the liver after 1 – 2 hours (Fig. 3). Such a scheme is in agreement with an earlier report that, after infusion of benfotiamine to the small intestine of the dog, mainly free thiamine (not S-benzoylthiamine) was detected in the carotid blood [35]. Free thiamine is not lipophilic and cannot cross the blood-brain barrier by simple diffusion. Transport of blood thiamine to the brain parenchyma is carrier-mediated and it is a slow process [37]. In the present study, we find that blood thiamine concentration in the control animals is approximately 0.4 μmol/l, a value close to the half-maximal activation constant for the high affinity transport of thiamine across the blood-brain barrier [8]. Though a second, low affinity, component of thiamine transport was also observed, its contribution was small. Thus, raising free blood thiamine concentrations does not necessarily lead to an important increase in thiamine transport across the blood-brain barrier. It is therefore not very surprising that benfotiamine administration does not lead to an increase in total thiamine content of the brain (Figs 2, 3 and 4). It should be noted however that one study showed a 90% increase of ThDP levels in the brains of rats that received a dose of 1645 mg of benfotiamine/kg of diet for 6 months [27].

### Differences between benfotiamine and lipophilic thiamine disulfide derivatives

Wada et al. already noted that benfotiamine was sparingly soluble in organic solvents such as benzene, chloroform and methanol, but was readily soluble in aqueous media at pH ≥ 8.0 [31]. This is not surprising as the phosphoryl group of benfotiamine has two negative charges at alkaline pH. Here, we confirm that benfotiamine is sparingly soluble in water at pH ≤ 7.0 and cannot be dissolved in octanol or oils. Thus benfotiamine should not be classified as a "lipophilic" compound as many authors still do [10,24,25,38]. Indeed, benfotiamine appears unable to diffuse across cell membranes. We have shown here that intracellular thiamine content is not increased in cultured neuroblastoma incubated in the presence of 10 μM benfotiamine, while it was increased ten-fold after incubation with 10 μM sulbutiamine [29]. Moreover, after a chronic treatment of rats with sulbutiamine intracellular thiamine derivatives were increased by respectively 250% (thiamine), 40% (ThMP), 25% (ThDP) and 40% (ThTP) [14].

This is in apparent contradiction with results obtained with cultured cells of endothelial origin [18-20,39], showing that benfotiamine is able to counteract glucose toxicity in these cells by increasing transketolase activity. However, the benfotiamine concentrations used were 50 – 100 μM, much higher than in our study. Hammes et al. even report that there was no effect on transketolase activity in cultured endothelial cells at 10 or 25 μM [18]. In any event, this is no proof that benfotiamine is able to cross the membranes: indeed, cultured endothelial cells seem to possess an ecto-alkaline phosphatase [40]. It is therefore likely that, in these cells, the added benfotiamine is at least partially dephosphorylated to S-benzoylthiamine that can enter the cells as in the case of the intestinal mucosa. The slow dephosphorylation to S-benzoylthiamine might also explain the lag period observed between the addition of benfotiamine to thiamine-depleted Neuro 2a cells and the increase in intracellular thiamine derivatives (Fig. 6). In erythrocytes, it was shown that fursultiamine, a lipophilic disulfide, is rapidly incorporated into the cells while benfotiamine is not [41]. Taken together, these results strongly suggest that benfotiamine is unable to cross plasma membranes unless it is dephosphorylated.

### Benfotiamine and sulbutiamine have different pharmacological profiles

Since the discovery of allithiamine [9], a number of derivatives were synthesized. These showed higher bioavailability than thiamine hydrochloride or mononitrate. Lipid-soluble thiamine derivatives were developed mainly in Japan for the treatment of beriberi. It was therefore surprising that sulbutiamine appeared to exert specific effects on brain function. Indeed, it seems to improve memory in rodents [42,43] and, in humans, it seems to be beneficial against functional asthenias [13,44,45]. These effects have not been reported with thiamine. This difference might be explained if we assume that sulbutiamine (or its degradation product thiamine disulfide) can cross the blood-brain barrier and have specific actions in neurons. However, there is so far no direct evidence that untransformed sulbutiamine is indeed found in the brain. Concerning benfotiamine, there is no evidence that it has any specific effect on the central nervous system, but during the last few years, there was considerable interest in the therapeutic potential of benfotiamine in peripheral tissues. Indeed, it was found effective for the protection of diabetic complications such as diabetic neuropathy [18-23] and alcoholic neuropathy [46]. Our results are in agreement with the different pharmacological profiles of sulbutiamine and benfotiamine. We previously found that sulbutiamine treatment significantly increases thiamine, ThMP, ThDP and ThTP content of rat brain [14], while the present results show that benfotiamine, at a twice higher dose, is unable to raise the levels of intracerebral thiamine phosphate derivatives (Figs 2, 3 and 4). This is in agreement with a previous study showing that after administration of ^3^H-benfotiamine, liver and kidney were labeled to a higher degree than brain and muscles [47], but this study did not make a difference between benfotiamine and its labeled metabolites and degradation products. Furthermore our results on cultured neuroblastoma cells show that benfotiamine, in contrast to sulbutiamine, does not easily cross cell membranes (Figs 5 and 6). It would therefore be interesting to test whether a thiamine disulfide compound such as sulbutiamine or fursultiamine, would not be more efficient and act at lower concentrations than benfotiamine in counteracting diabetic complications. A recent study has shown that, at high concentration (300 μM), benfotiamine exerts a direct antioxidant effects in three different kidney cell lines, independently of its transformation in thiamine and increased transketolase activity [48]. It is however not yet clear to what extend, if any, this may be involved in the improvement of diabetic complications.

## Conclusion

Our results show that oral administration of benfotiamine leads to significant increases in thiamine, ThMP and ThDP levels in blood, liver but not in the brain. This difference is in agreement with the known pharmacological profile of benfotiamine, i.e. the beneficial effects of the drug concern peripheral tissues but not the central nervous system. Like disulfide derivatives, benfotiamine may be useful for the treatment of acute peripheral syndromes of thiamine deficiency because it is better absorbed than thiamine, but in contrast to sulbutiamine, it seems to be devoid of specific effects on brain function. On the other hand, sulbutiamine should be much more efficient than benfotiamine in the treatment of Wernicke-Korsakoff syndrome. Physico-chemical data as well as studies with isolated cells strongly suggest that, in contrast to disulfide derivatives, benfotiamine is not a lipophilic compound and is unable to diffuse through cell membranes unless it is first dephosphorylated by ecto-alkaline phosphatases. Finally, it seems important to us that, because of different chemical, metabolic and pharmacological properties, a clear distinction should be made between lipophilic thiamine disulfides (derived from allithiamine) and S-acyl derivatives such as benfotiamine.

## Methods

### Administration of benfotiamine

Benfotiamine (Sigma-Aldrich) was dissolved in a 200 mM solution of hydroxypropyl-β-cyclodextrin (HP-β-CD, Roquette, Lestrem, France) at a concentration of 25 mg/ml. The solution was homogenized at 4°C. The mice were force-fed with a dose of 100 mg benfotiamine/kg in approximately 100 μl using a syringe prolonged by a flexible tube that was inserted into the stomach. The mice strains (male, 8 weeks old) were either C57B6 or Sv129. Control animals received an oral administration of 100 μl HP-β-CD solution (200 mM) without benfotiamine. The local committee for animal care and use approved all animal experiments.

### Sample preparation and determination of thiamine derivatives by HPLC

The mice were anesthetized with isoflurane and the forebrain and the liver were collected. For collecting blood samples, the animals were anesthetized with isoflurane and heparin sulfate sodium salt (5000 I.U./ml, Leo Pharma, Wilrijk, Belgium) was injected directly into the heart (50 μl). Tissues were stored at -80°C until sample preparation. About 50 mg of tissue were homogenized in 500 μl trichloroacetic acid (TCA, 12% w/v) in a glass-glass homogenizer. The homogenates were centrifuged (5000 × g, 10 min, 4°C) and the TCA was extracted from the supernatant with 3 × 1.5 ml diethylether. The samples were kept at -20°C until use. HPLC analysis was performed exactly as previously described [49]. Protein concentrations were determined by the method of Peterson [50].

### Cell culture

Mouse neuroblastoma cells (Neuro 2a) were grown as previously described in 100 mm Petri dishes containing 10 ml Dulbecco's modified Eagle's medium (DMEM, N.V. Invitrogen SA, Merelbeke, Belgium), supplemented with 10% fetal calf serum [29]. When the dishes were nearly confluent (5 – 10 mg of protein/dish), the culture medium was replaced by 10 ml of saline (145 mM NaCl, 5 mM KCl, 1 mM MgCl_2_, 1 mM CaCl_2_, 10 mM glucose, 10 mM Hepes-Tris, pH 7.3) containing either 10 μM benfotiamine or 10 μM thiamine. A 10 mM stock solution of benfotiamine was prepared in a mixture of ethanol (50%) and water. HCl was added until a homogenous solution was obtained. After incubation (0 to 4 hours at 37°C), the cells were centrifuged (2000 × g, 2 min) and the pellet was suspended in 500 μl TCA (12%). The proteins were sedimented (5000 × g, 3 min) and thiamine derivatives were determined in the supernatant after extraction of TCA by diethylether as described above.

Neuro 2a cells were thiamine-depleted as previously described [8,30]. Briefly the cells were grown in DMEM medium devoid of thiamine (Invitrogen). The concentration of fetal calf serum that contains about 14 nM of thiamine, was reduced to 5%. The medium was replaced every 2 days and the cells subcultured every 4 days. After 8 days, either thiamine (1 μM) or benfotiamine (1 μM) were added and the intracellular thiamine levels were determined as a function of time (up to 6 h) as described above.

### Statistics

Data were analyzed by multivariate analyses of variance (MANOVA), followed by ANOVA for follow-up comparisons or two-way ANOVA followed by the Bonferroni post-test.

## Abbreviations

AThTP: adenosine thiamine triphosphate; DMEM: Dulbecco's modified Eagle's medium; HP-β-CD: hydroxypropyl-β-cyclodextrin; TCA: trichloroacetic acid; ThMP: thiamine monophosphate; ThDP: thiamine diphosphate; ThTP: thiamine triphosphate.

## Competing interests

The authors declare that they have no competing interests.

## Authors' contributions

M–LV and SS performed most of the experimental part of the work. MG contributed to the experimental part. MP and BE suggested to formulate benfotiamine with cyclodextrins and made the initial solubility tests. J–CP and LB were the project leaders and participated in the design. LB wrote the final manuscript. All authors read and approved the final manuscript.
